# Outcome of ileostomy performed among emergency cases: A prospective cross-sectional study in India

**DOI:** 10.6026/973206300214255

**Published:** 2025-11-15

**Authors:** Harendra Kumar, Rekha Kumari, Anil Kumar, Deepak Kumar

**Affiliations:** 1 Department of Trauma Surgery and Critical Care, All India Institute of Medical Sciences, Patna, India

**Keywords:** Ileostomy, perforation, peritonitis, emergency

## Abstract

Ileostomy is a life-saving procedure often performed in emergency settings for complicated cases such as ileal perforation, particularly due to enteric fever and intestinal tuberculosis. We conducted a prospective study between October 2018 and September 2020, including 57 patients who underwent temporary ileostomy for faecal diversion in emergency laparotomies. The majority of patients were male, with common presenting symptoms being abdominal pain, fever and vomiting. Postoperative complications were frequent, the most common being skin excoriation and ileostomy diarrhoea, while the overall mortality rate was 3.51% and the average hospital stay was 10-14 days. Despite these challenges, temporary ileostomy remains an effective and essential intervention in critically ill patients, with manageable complications and favourable outcomes.

## Background:

Ileostomies play a crucial role in mitigating morbidity and mortality associated with anastomotic leakage in colonic and small gut anastomoses, particularly in cases of septicemic patients with ileal perforation stemming from various causes such as typhoid fever, tuberculosis, trauma, or ruptured appendix [1]. Despite their life-saving potential, ileostomies are not without their own set of complications, which can arise in up to 16.9% of cases within 60 days post-operation [2]. These complications include stomal obstruction, skin excoriation and dehydration resulting from high ileostomy output. Additionally, the creation of a diverting ileostomy during surgical treatment, while associated with morbidity, is often indispensable in preventing further life-threatening complications. Complications stemming from ileostomies range from lacerations to stoma necrosis, stoma prolapse and bowel obstruction, with the latter often necessitates surgical intervention [3]. Our study aims to investigate the outcome and complications in our tertiary care centre, where a significant number of late-presenting acute peritonitis cases are managed through ileostomy procedures. This study is in line with STROCSS guidelines [4]. Therefore, it is of interest to report the outcome of ileostomy performed among emergency cases in India.

## Materials and Methods:

This prospective study was conducted at the surgical emergency department over a period spanning from October 2018 to September 2020. This study was done as per the Institutional Ethical Committee norms. The institute ethics committee approved the study; vide letter number AKU/PMCH/IEC/24/2207. The patients were enrolled in the study as per the following inclusion and exclusion criteria.

## Inclusion criteria:

[1] All cases requiring emergency laparotomy, excluding those falling under the exclusion criteria, necessitating ileostomy as a protective fecal diversion.

[2] Only cases involving temporary ileostomies were included.

## Exclusion criteria:

[1] Cases where ileostomies were performed elsewhere.

[2] Permanent end ileostomies for malignant diseases.

[3] Patients below 12 years of age.

[4] Patients requiring urinary diversion procedures involving the creation of an intestinal stoma.

[5] Patients presenting with biochemical and physiological complications.

All patients presenting to the surgical emergency department with features of acute abdomen and meeting the aforementioned criteria were enrolled. Detailed clinical evaluations were conducted by the surgical team, capturing demographic data, co-morbidities, therapeutic interventions, pre-operative assessments, intraoperative findings, postoperative hospital course and follow-up information. Clinical histories and vital signs were meticulously recorded, along with systemic examinations and routine hematological, biochemical and radiological investigations. Following resuscitation, patients underwent exploratory laparotomy within 24 hours of hospital admission under general anesthesia. Intraoperative procedures included a thorough survey of the peritoneal cavity, noting findings such as peritoneal fluid type, intestinal perforations, bands, strictures and adjacent bowel conditions. During ileostomy creation, care was taken to ensure the stoma was positioned sufficiently above the skin level to minimize skin excoriation. Post-operatively, patients were closely monitored for complications such as wound infections, dehiscence, fecal fistulae, stoma-related issues, mortality and duration of hospital stay. All data were analysed using IBM SPSS Statistics for Windows, Version 25.0. (Armonk, NY:IBM Corp.).

## Results:

A total of 57 patients of ileostomy were included in the study. The age of patients studied ranged from 18 to 75 years with mean being 39.77±16.37 years.Six patients (10.53%) were age 13 to 20 years, 14 (24.56%) were age 21 to 30 years, 16 (28.07%) were age 31 to 40 years and seven patients (12.28%) were age 41 to 50 years. Figure 1 (see PDF) presents the male to female ratio (41: 16). Among the 57 cases, 44 patients presented with pain abdomen associated with fever and vomiting. The commonest sign were abdominal tenderness (89.47%), followed by guarding and rigidity (87.72%), absence of bowel sound (50.88%), Shock (36.84%), Dehydration (33.33%), abdominal distension (49.12%) and obliteration of liver dullness (29.82%). Following admission, clinical evaluation and resuscitation, patients were subjected to routine and specific investigations. 41 patients had gas under diaphragm and 16 patients had multiple air fluid level in erect abdomen X-rays. 19 patients underwent USG whole abdomen, out of which intra-peritoneal free fluid was present in 16 patients and absent in 3 patients. On exploration, gas come out on opening the peritoneum in 35 cases. There was gross contamination of peritoneal cavity in most cases. Peritoneal cavity was found to contain copious amount of faecopurulent material. In most of the cases peritoneal cavity was filled with feculent or purulent fluid followed by predominantly haemorrhagic and clear fluid. Most of the patients on exploration had bowel perforation followed by stricture and gangrenous bowel segment depicted in Figure 2 (see PDF). The most common indication of ileostomy was hollow viscus perforation due to enteric fever, intestinal tuberculosis, non-specific and firearm/penetrating injuries followed by blunt trauma abdomen (Figure 3 - see PDF).

The age group wise segregation of different pathologies / indications revealed high number of enteric perforation patients in age group 21-30 and 31-40 years accounting for 54.55% of total enteric perforation cases. Similarly the incidence of intestinal tuberculosis, firearm / penetrating injuries and blunt trauma abdomen and non -specific were also higher in age group 21-30 and 31-40 years accounting for 40%, 50%, 83% and 45% respectively. In the study population 38.6% of all perforation was enteric in origin followed by17.54% of intestinal tuberculosis, and 14.04% in firearm / penetrating injury. 50% of all strictures were tubercular in origin and remaining 50% were of non-specific inflammation histopathologically. Gangrene was encountered in 37.5% of all blunt trauma abdomen patients resulting due to mesenteric tear resulting to devascularisation of bowel segment. Gangrene due to non- specific bowel segment ischemia was encountered in 50% cases. One patient had non-specific inflammatory stricture resulting into obstruction and proximal perforation. Most common type of ileostomy done was loop followed by double barrel and end ileostomy (Figure 4 - see PDF). Post-operative complications were found in varying proportions in most of the patients. The overall rate and incidence of complications is detailed in Table 1 (see PDF) and Figure 4 (see PDF). The patients were divided into three groups based on the time from creation of ileostomy to closure (Table 2 - see PDF). There was no statistically significant difference among the groups.

## Discussion:

Although the first stomas were described and constructed in the 19th century, they were associated with innumerable complications and hence did not establish themselves as a favourite procedure amongst surgeons as well as patients. With the advent of better surgical techniques, asepsis and post-operative care, the traditional complications were associated with much lesser morbidity. The purpose of bringing the ileum over abdominal wall via surgical opening is to evacuate stool from the body via the stoma instead of the natural route which is anus. The stoma is indicated mainly to protect a distal anastomosis after perforation, to evacuate stool from the body after surgery in case of Crohn's disease, ulcerative colitis and familial polyposis and to relieve bowel obstruction [5]. In our study, most of the patients belonged to two age groups 21-30 years and 31-40 years, comprising of 24.56% and 28.07% respectively of total cases. The high incidence of cases in the above age group could be attributed to the incidence of enteric perforation, intestinal tuberculosis, non-specific inflammatory bowel diseases and blunt trauma/penetrating injuries. Stoma creation in different age group was reported by many studies like Aziz *et al.* [6] (24-48 years), Massenga *et al.* [7] (10-70 years), Qassim *et al.* [8] (48.25 to 67 years). In our study, majority (72%) of the patients were male and 28% were female, similar to previous studies by Ahmad *et al.*, Pandiaraja *et al.*, Rajput *et al.*, and Chaudhary *et al.* which also reported a higher proportion of male patients undergoing stoma formation [9, 10, 11-12]. In our study, the Common presenting symptoms were pain abdomen (96.49%), fever (64.91%) and vomiting (52.63%). The commonest sign were abdominal tenderness, guarding and rigidity, absence of bowel sound and Shock. Ansari *et al.* also reported the similar findings [13]. Obliteration of liver dullness was seen in 29.82% of patients and absent bowel sounds in 50.88% of patients. Among 57 patients, 21 (36.84%) patients presented to the emergency room in shock. Defunctioning ileostomy was created in 57 patients and the most common indication was for the management of hollow viscous perforation (35 patients). In remaining 22 patients either stricture of the bowel segment or gangrenous bowel segment was present. The most common aetiology of perforation was enteric fever (38.60%, n=22) followed by tubercular (17.54% n=10) and firearm / penetrating/ blunt abdominal injury (14.04% n=8). The results were similar with result of study done by Ahmad *et al.* (Enteric fever-38%, Tuberculosis-18%), Ahmad *et al.* (Enteric fever-81.13%, Tuberculosis-9.43%) and Rajput *et al.* (Enteric fever- 66%, Iatrogenic-10.70%) [9, 11]. Although the site of the perforation varied, the intra-operative findings warranted a stoma in these patients. Loop ileostomy was the most commonly performed surgery (84.21% n=48) amongst patients who presented with hollow viscous perforation as the perforation was most commonly located in the ileum or the base of appendix. Other procedures were double barrel ileostomy (10.52% n=06) and end ileostomy (5.26% and n=03). The results are similar with findings of previous study done by Ahmad *et al.* in which loop ileostomy were performed in 84.20% followed by double barrel ileostomy (5.3%) and end ileostomy (3.9%) [9, 14, 15- 16].

In our study, 80% of patients developed stoma-related complications which are similar to study done by Pandiaraja *et al.* [10]. Among the complications, skin excoriation (38.84%) is a more common complication, followed bystomaldiarrhea (22.80%) and laparotomy wound infection (17.54%). Other less common important complications were dyselectrolytemia, ileostomy prolapse, ileostomy retraction, ileostomy stenosis, parastomal hernia, obstruction and anastomotic leak following stoma closure. The complication rate observed in our study was comparable to that reported in earlier studies. Ahmad *et al.* observed skin excoriation in 36% and wound infection in 13% of cases, while Rajput *et al.* reported skin excoriation in 21.4% of patients and poor siting of stoma in 10.7% [9, 11]. Similarly, Dawes & Gahagan *et al.* also noted skin complications and retraction as the most frequent stoma-related problems in their large cohort study [17]. In the present study, most of the complications were managed conservatively with stoma site care provided by trained professionals, but few complications like stomal retraction and intestinal obstruction necessitated surgical intervention. The stoma related skin complications may be attributed to poor stoma care amongst the patient population. The proper preoperative planning, intraoperative decision by senior consultant, postoperative stoma care and early reversal of stoma consideration reduce the postoperative complications as per many studies [18- 19]. In the present study the mortality rate was 3.53% which is higher as compared to 1.5% in a study done by Poskus *et al.* [20]. Hospital stay for most of the patients was between 10-14 days (mean = 39.77±16.37). Patients with longer stay were those who had surgical site infection and/or peristomal ulceration. The average hospital stay was 10-14 days in present study as compared to 8 days in other study [21]. Patients with longer stay were those who had surgical site infection and/or peristomal ulceration. Ileostomy is a state of social and psychological trauma for the patient due to visible fecal waste in the bag and its smell. It has an adverse effect on the quality of life and derails the professional life as well. Few patients suffer from psychological symptoms. The symptoms worsen with the occurrence of ileostomy related complications like skin excoriation. In this study 10.53% (n=6) patients had psychological symptoms in the form of depression, stopped speaking/eating properly. The psychosocial issues are the major concern in post-operative period suggested by other study also [22]. All these symptoms gradually improved with time as the ileostomy matured and after they were explained about restoration of normal life within short span after the closure of stoma. Most of the patients during the waiting period for second surgery were able to lead a normal social and routine life but they missed their work as they found it difficult to work with the stoma.

## Limitations of the study:

[1] Limited follow-up period: The one-year follow-up period may not capture long-term complications or outcomes beyond this timeframe, potentially underestimating the overall impact of ileostomy.

[2] Small sample size: With only 57 patients included in the study, generalizability to broader populations may be limited and there's a risk of insufficient statistical power to detect less common complications or trends.

[3] Selection bias: Exclusion criteria, such as excluding patients with end ileostomy for malignancy and specific complications, may introduce selection bias and limit the study's applicability to all ileostomy patients.

[4] Data collection reliance: Depending solely on patient records and self-reporting for data collection may introduce information bias or missing data, potentially affecting the accuracy and completeness of the findings.

[5] Single-center study: Conducting the study in a single surgical emergency department may limit the diversity of patient populations and treatment approaches, reducing the external validity of the results and limiting generalizability to other healthcare settings.

## Conclusion:

Temporary protective ileostomy is preferred for fecal diversion, especially in critically ill patients. Common indications include enteric fever and tuberculosis. Complications like skin excoriation and prolapse require careful management. Despite challenges, ileostomy remains life-saving with manageable complications, emphasizing its temporary nature.

## Financial Support: 

Nil
